# T2-Pseudonormalization and Microstructural Characterization in Advanced Stages of Late-infantile Metachromatic Leukodystrophy

**DOI:** 10.1007/s00062-020-00975-2

**Published:** 2020-11-23

**Authors:** Pascal Martin, Gisela E. Hagberg, Thomas Schultz, Klaus Harzer, Uwe Klose, Benjamin Bender, Thomas Nägele, Klaus Scheffler, Ingeborg Krägeloh-Mann, Samuel Groeschel

**Affiliations:** 1 Department of Neurology and Epileptology, Hertie Institute for Clinical Brain Research, University of Tübingen, Tübingen, Germany; 2 High Field Magnetic Resonance, Max-Planck Institute for Biological Cybernetics, Tübingen, Germany; 3 Biomedical Magnetic Resonance, University Hospital, Tübingen, Germany; 4 B-IT and Institute of Computer Science, University of Bonn, Bonn, Germany; 5 Department of Neuropediatrics, University Children’s Hospital, Tübingen, Germany; 6 Department of Diagnostic and Interventional Neuroradiology, University Hospital Tübingen, Tübingen, Germany

**Keywords:** NODDI, Myelin water imaging, Diffusion kurtosis imaging, Magnetization transfer ratio

## Abstract

**Purpose:**

T2-weighted signal hyperintensities in white matter (WM) are a diagnostic finding in brain magnetic resonance imaging (MRI) of patients with metachromatic leukodystrophy (MLD). In our systematic investigation of the evolution of T2-hyperintensities in patients with the late-infantile form, we describe and characterize T2-pseudonormalization in the advanced stage of the natural disease course.

**Methods:**

The volume of T2-hyperintensities was quantified in 34 MRIs of 27 children with late-infantile MLD (median age 2.25 years, range 0.5–5.2 years). In three children with the most advanced clinical course (age >4 years) and for whom the T2-pseudonormalization was the most pronounced, WM microstructure was investigated using a multimodal MRI protocol, including diffusion-weighted imaging, MR spectroscopy (MRS), myelin water fraction (MWF), magnetization transfer ratio (MTR), T1-mapping and quantitative susceptibility mapping.

**Results:**

T2-hyperintensities in cerebral WM returned to normal in large areas of 3 patients in the advanced disease stage. Multimodal assessment of WM microstructure in areas with T2-pseudonormalization revealed highly decreased values for NAA, neurite density, isotropic water, mean and radial kurtosis, MWF and MTR, as well as increased radial diffusivity.

**Conclusion:**

In late-infantile MLD patients, we found T2-pseudonormalization in WM tissue with highly abnormal microstructure characterizing the most advanced disease stage. Pathological hallmarks might be a loss of myelin, but also neuronal loss as well as increased tissue density due to gliosis and accumulated storage material. These results suggest that a multimodal MRI protocol using more specific microstructural parameters than T2-weighted sequences should be used when evaluating the effect of treatment trials in MLD.

## Introduction

Metachromatic leukodystrophy (MLD) is an autosomal recessive inherited lysosomal storage disease caused by the deficiency of the lysosomal sulfatide degrading enzyme arylsulfatase A. The resulting accumulation of sulfatides in the central nervous system leads to the dysfunction and progressive destruction of microglia and oligodendrocytes [1, 2]. This gives rise to a progressive demyelination as the main driver of the pathology leading to impaired motor and cognitive abilities [3–5]. As a correlate of demyelination, magnetic resonance imaging (MRI) shows early changes in the white matter in the form of T2 hyperintensity, which spreads from periventricular to bi-hemispheric regions [6–8]. Particularly in the late-infantile type, which begins within the first 2 years of life, there is a relatively uniform pattern of spread parallel to the loss of motor function [7]. In addition, the quantification of T2 signal hyperintensity in the form of a demyelination load was found to show good correlation with motor and cognitive symptoms in the late-infantile [9] and juvenile types [10, 11].

While MRI has been used mainly as a diagnostic tool early in the disease course, there are usually no MRIs available in the advanced disease stage, especially in the late-infantile form where there is severe and rapid disease progression; however, with new therapeutic options currently being evaluated in clinical trials in children with the late-infantile form of the disease [12], there is high interest in understanding brain changes in the late disease stage due to the homogeneously rapid neurological course and the lack of placebo-controlled trials in these severe rare diseases.

Therefore, it is necessary to assess cerebral tissue changes throughout the whole clinical course using MRI sequences, which can characterize and quantify white matter microstructure [13, 14] to provide a better understanding of pathophysiological mechanisms and treatment responses. For this purpose, diffusion tensor imaging has already been used and due to temporal changes of axial diffusion in the corpus callosum over the course of the disease, it has been presumed that there is also an additive axonal component after initial dominant myelin loss [15]. This would be consistent with experimental data, which also described an accumulation of sulfatides and the resulting functional restriction in neurons [16, 17]; however, diffusion tensor parameters are limited in their informative value, especially in the white matter due to crossing fibers [18] and are often not specific [19]. More recent sequences for further characterization of microstructural changes and (de)myelination processes are available including diffusion-weighted imaging including NODDI model, myelin water fraction (MWF), magnetization transfer ratio (MTR), effective transverse relaxation rate R2*, T1-mapping and quantitative susceptibility mapping in addition to the already implemented MR spectroscopy [20].

We applied these sequences in a multimodal MRI protocol to children with late-infantile type MLD in the advanced stage of the natural disease course so to investigate their principal benefit regarding MRI changes in metachromatic leukodystrophy. These patients were of special interest because they showed intriguing findings with areas of normal T2 intensities in the white matter despite the late stage of the disease and thus contradicted the aforementioned current understanding of the MRI course of the disease.

## Methods

### Subjects

Patient data were collected as part of a natural history study of the German leukodystrophy network Leukonet [7]. We included 26 patients with late infantile type MLD in the study to expand on previous investigations of T2-hyperintensities of untreated patients covering a larger age range [7, 9]. Of the 26 patients 6 had an MRI follow-up scan. The median age at imaging was 2.25 years (minimum 0.5, maximum 5.17 years), and median follow-up time was approximately 27 months (795 days, range 252–1918 days) and 13 were female. MLD was diagnosed as deficiency of arylsulfatase A (ASA) together with an increase in urinary sulfatide level and/or pathogenic mutations in the MLD gene, together with typical clinical features. Age at onset for late-infantile MLD was defined as 30 months and younger [5]. The study was approved by the ethical committees of the University of Tübingen, Germany. Written informed consent was given by the parents. Only data with sufficient quality for volumetric analysis were included.

Healthy subject data for the advanced sequence parameters were acquired and analyzed in advance to establish a control cohort that consisted of 21 healthy subjects developing typically (median age 14.9 years, range 9–40 years, 11 females).

### MRI Acquisition

The MRI sequences of 23 patients were acquired on 1.5T and 3 T scanners and consisted of conventional clinical routine images with a high-resolution T1-weighted sequence (magnetization prepared rapid gradient echo sequence with voxel size typically 1 × 1 × 1 mm) and a T2-weighted axial sequence (spin-echo sequence with an echo time/repetition time 99/5940 ms, voxel size 0.78 × 0.78 × 4 mm) [7, 9].

Three patients in the late stage of the disease were additionally examined in a follow-up scan using a 3T scanner (Skyra or Prisma, Siemens Healthineers, Erlangen, Germany) with an extended MR protocol, as done for the control cohort, explained in detail elsewhere [13]. Please note that not all sequences were acquired in all patients and controls. In brief, the sequences included:

- Axial T2-weighted turbo-spin echo (TR/TE: 10810/84 ms, voxel size 0.49 × 0.49 × 3 mm^3^) (*n* = 20 controls, 23 patients).
- MPRAGE (TR/TE/TI: 2300/4.11/900 ms, flip angle 9°, voxel size 1 × 1 × 1 mm^3^) (*n* = 20 controls, 23 patients).
- MP2RAGE (TR/TE/TI1/TI2: 4000/3.04/700/2500 ms, flip angle 4/5°, for the first and second inversion, respectively, voxel size 1 × 1 × 1 mm^3^) (*n* = 2 controls, 2 patients).
- Diffusion-weighted (DW) images using a high angular resolution, twice-refocused spin echo planar imaging sequence (TR/TE: 9100/89 ms; voxel size 2 × 2 × 2 mm^3^). Diffusion weighting gradients were applied in 64 directions with b = 2000 s/mm^2^ and 30 directions with b = 700 s/mm^2^. For distortion correction, an additional image without diffusion weighting was acquired with the same resolution, FOV and readout band width, but with reversed polarity of the phase-encoding gradients. *N* = 20 controls, 3 patients.
- MR spectroscopy using a chemical-shift imaging (CSI) sequence with the same axial orientation as the axial T2-weighted image positioned above the lateral ventricles (TR/TE: 1600/135 ms, voxel size 5 × 5 × 15 mm^3^, FOV: 160 × 160 mm^2^; 16 × 16 matrix, interpolated to 32 × 32). *N* = 11 controls, 3 patients.
- Myelin water fraction (MWF) imaging, using a Carr-Purcell-Meiboom-Gill (CPMG) sequence modified to have shorter radiofrequency (RF) pulse durations and increased band width of the 180° RF pulse. (TR/TE_1_: 3000/10 ms, TE_2_/../TE_32_: 20 ms/../320 ms in steps of 10 ms, voxel size: 0.75 × 0.75 × 5 mm^3^, FOV: 192 × 192 mm^2^; 128 × 128 matrix, slice thickness 5 mm, 1 slice). In addition, a B_1_ map was acquired from the same slice and used for MWF quantification. *N* = 19 controls, 3 patients).
- Magnetization transfer ratio (MTR) images (using two sets of balanced steady state free precession 3D images with slab-selective RF excitation pulses, TR1/TE1: 4.23/2.115 ms for the set with 1.5 ms duration of the RF pulses, and TR2/TE2: 2.93/1.465 ms for the MT-weighted set with 0.2 ms pulses. The other sequence parameters were fixed with a reconstructed voxel size: 0.65 × 0.65 × 1.3 mm^3^; FOV: 256 × 256 mm^2^, 384 × 384 matrix, 144 partitions, flip angle of 20° (19 controls, 3 patients.
- 3D flow-compensated gradient echo for quantitative mapping of the effective transverse relaxation time (R2*) and quantitive susceptibility maps (QSM) at eight different echo times (TR/TE1..TE8: 50/4.5/10/15/21/27/33/39/46 ms, flip angle of 15° and a voxel size of 1 × 1 × 2 mm^3^. *N* = 5 controls, 2 patients.

### Image Processing

Image processing and data analysis were performed as previously described [9, 13].

As mentioned before, to quantify the volume of T2 hyperintense white matter, the demyelination load was measured in all 26 patients using multispectral segmentation of high-resolution T1-weighted and axial T2-weighted images [11, 21]. The ratio of the demyelination load and total WM volume was calculated [9].

Correction for motion, EPI distortions and/or variation in signal intensity was applied to the DWI data set [13]. Maps of mean diffusivity (MD), fractional anisotropy (FA), as well as axial and radial eigenvalues, were calculated using the standard log-linear least squares fit of the tensor model to the subset of diffusion-weighted images with b = 0 and b = 700 s/mm^2^ [22, 23]. Axial diffusivity (AD) was calculated from the axial eigenvalue, radial diffusivity (RD) from the average of the two radial eigenvalues. Maps of mean, axial, and radial kurtosis (MK/AK/RK) were computed from a fit of the diffusional kurtosis model using quadratic cone programming with semi-definiteness constraints on the diffusivities [13]. Moreover, NODDI-derived parameters, including intracellular volume fraction (ICVF), Isotropic volume fraction (ISO) and orientation dispersion (ODI) were calculated based on a two-compartment model [24].

MRI spectra were analyzed quantitatively using the LCModel [25]. Absolute concentrations of N‑acetylaspartate and N‑acetylaspartylglutamate (NAA), creatine and phosphocreatine (Cr), as well as choline-containing compounds (Cho) were calculated to further analyze ratios of NAA/Cr and Cho/Cr.

For MWF all parameters including the flip angle, were fitted [26]. In the second step, the flip angle was fixed to the voxel-specific value measured by the B1 fitted with a 4th order polynomial and scaled to match the median B1 value across the slice found in the first step [27]. Number of fitted T2 points: 64; Chi^2^ regularization of 1.02, range of T2-values for the myelin water: 15–40 ms and for the remainder: 40–200 ms.

The MT ratio (MTR) map was calculated in percentage units from the co-registered non-MT and MT images using the following equation: (non-MT − MT) / non-MT.

For QSM the frequency maps were fitted by using the phase unwrapping and multi-echo fitting tool from the MEDI-Toolbox [28] and the BET masking tool from FSL [29]. Background dipole field modulations were removed with RESHARP [30] and the local dipole inversion process was performed by using the fast magnitude-weighted L1-regularization technique with total variation penalty [31].

For each subject, conventional images, MWF, MTR, and QSM were all co-registered (rigid body transformation using normalized mutual information) and interpolated to the subject’s FA map; therefore, it was made sure that all image modalities per subject were transformed into the same space and image dimensions for further analysis. Images were processed and analyzed using MRtrix (version 0.3.12, www.github.com/MRtrix3/mrtrix3 [32]), Matlab (version R2014b, www.mathworks.com) and FSL tools (version 5, http://fsl.fmrib.ox.ac.uk/fsl/fslwiki/FSL, [33]).

**Fig. 1:**
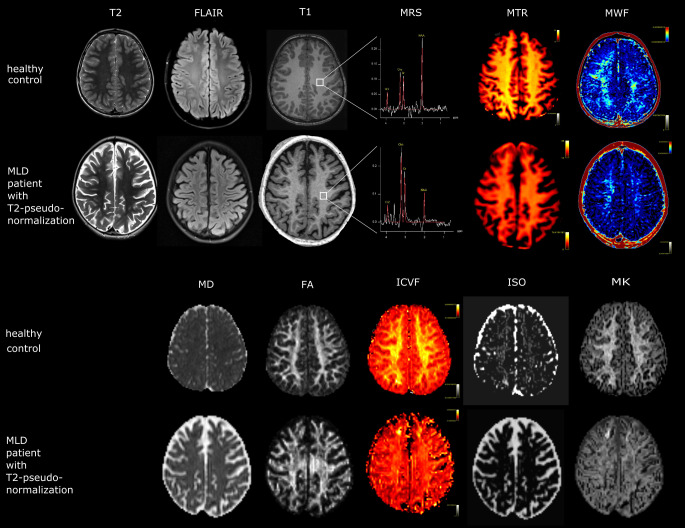
The applied sequence parameters are visualized while results of an each exemplary control and patient with pseudonormalization stand vis à vis. T2, FLAIR and T1 show no visible signal alterations, while differences in MR spectroscopy (MRS), magnetization transfer ratio (MTR), myelin water fraction (MWF), mean diffusivity (MD), fractional anisotropy (FA), qisotropic volume fraction (ISO), intracellular volume fraction (ICVF) and mean kurtosis (MK) are evident

Fig. 1 provides an overview of the different image parameters and their evaluation.

### Data Analysis

Demyelination load (as a ratio to total WM volume) was correlated with the patient’s age and a regression analysis was performed using a quadratic regression model to estimate the mean change over time and its 95% confidence intervals using R (ggplot2, www.r-project.org).

For all parameters, data evaluation was performed using a region of interest (ROI) in the centrum semiovale within the corticospinal tract (CST-CS). The CST was delineated using tractography based on constrained spherical deconvolution. The ROI was set manually within the level of the CSI and MWF slice, above the level of the lateral ventricles, as done before [13].

The values of the ROIs were determined in both hemispheres of the brain (symmetrical disease burden was assumed) and the mean value and standard deviation of the corresponding ROI per group (3 patients and 20 healthy controls) were calculated. A statistical analysis was conducted using the Mann-Whitney U‑test comparing the group level results between the ROI locations of the patient and control group, while *p*-values <0.05 were considered as significant.

As this study was considered exploratory with a relatively small sample size, the *p*-values for these analyses were not corrected for multiple comparisons and can, therefore, be regarded as descriptive.

## Results

**Fig. 2:**
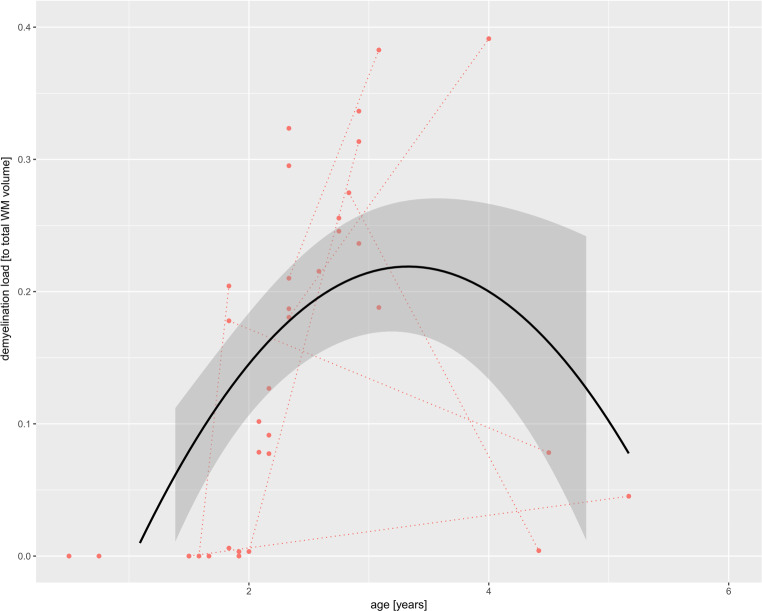
The demyelination load depending on the patient age shows a parabola-shaped course with rapidly increasing amount of T2-hyperintensities of white matter (WM) in the early stages of the disease yet decrease in the advanced stages to normal T2 intensity values (pseudonormalization). Patients that are measured several times are highlighted by *lines* connecting the different measurement time points. The *black line* indicates the median course of all patients included and the area in *grey* shows the standard deviation

The determined demyelination load depending on the patient’s age is shown in Fig. 2. Demyelination load increased rapidly and homogeneously beginning at the age of around 1.5 years, reaching a maximum at around 3–4 years but declined thereafter. Interestingly, there were no T2-hyperintensities before the age of ~1.75 years, and almost normal levels again in the 3 patients after the age of 4 years.

**Fig. 3:**
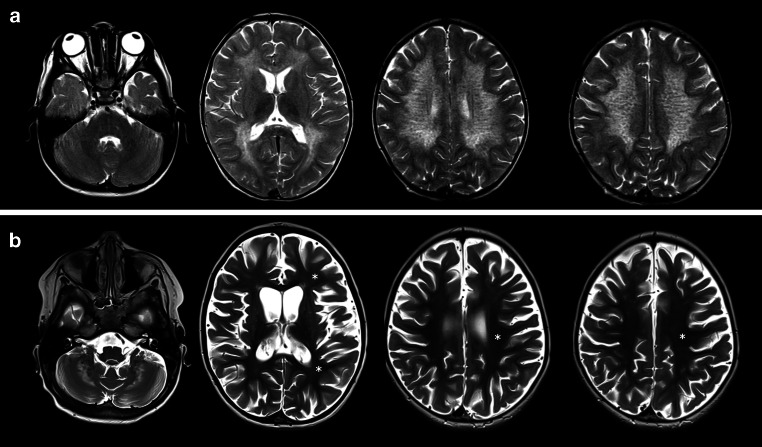
Example for T2-pseudonormalization: characteristic T2-hyperintensities of white matter (WM) are shown in the MRI of one exemplary patient at the time of diagnosis with the age of 2.8 years (**a**). In a follow-up scan at the late stage of the disease with 4.4 years (**b**), T2 hyperintensities of supratentorial WM are barely evident aside from WM in the cerebellum. *Asterisk* T2-pseudonormalization

Fig. 3 shows MRI results from one of the 3 patients with the most pronounced reduction of the demyelination load. The first MRI at the age of 2.8 years shows the characteristic WM changes with symmetrical T2-hyperintensities. At the age of 4.4 years, these changes are barely detectable within the supratentorial WM, signal abnormalities can still be detected only in the cerebellum; however, a severe cerebral atrophy can be observed at the late stage.

**Fig. 4:**
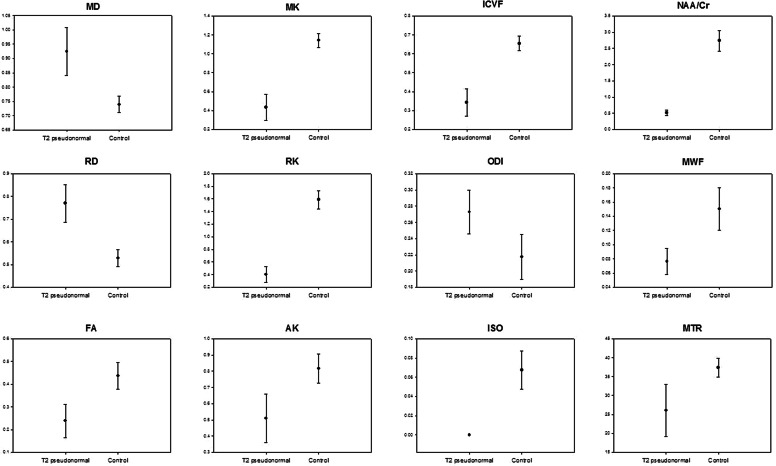
Mean values and standard deviations of the different quantitative MRI parameters that were found to be significantly different in pseudonormal T2 compared to the control group, measured in the corticospinal tract at the level of the centrum semiovale

In the 3 patients with a demyelination load reduction or stable WM conditions multimodal imaging was supplemented to better characterize the microstructure of the WM. Fig. 4 shows the parameters with statistically significant differences visualized in comparison to the values of the healthy controls.

The raw values can be found in Table 1.

**Table 1:** Mean values and standard deviations of the different quantitative MRI parameters acquired in patients with pseudonormal T2 and control in an ROI in the white matter (WM) of the corticospinal tract (CST) at the level of the centrum semiovale (CST-CS)

Parameter	CST-CS WM						
	Pseudonormal T2			Control			
	*n*	Mean	SD	*n*	Mean	SD	*p*-value
							(Mann-Whitney *U* test)
MD [mm^2^/s]	3	0.92	0.08	20	0.74	0.03	**0.001**
FA	3	0.24	0.07	20	0.44	0.06	**0.001**
AD [mm^2^/s]	3	1.22	0.08	20	1.17	0.10	0.354
RD [mm^2^/s]	3	0.77	0.08	20	0.53	0.04	**0.001**
MK	3	0.44	0.14	20	1.14	0.07	**0.001**
RK	3	0.40	0.12	20	1.59	0.15	**0.001**
AK	3	0.51	0.15	20	0.82	0.09	**0.002**
ICVF	3	0.34	0.07	20	0.65	0.04	**0.001**
ODI	3	0.27	0.03	20	0.22	0.03	**0.008**
ISO	3	0.00	0.00	20	0.07	0.02	**0.001**
NAA/Cr	3	0.51	0.09	11	2.74	0.32	**0.005**
Cho/Cr	3	0.25	0.02	11	0.23	0.03	0.368
MWF	3	0.08	0.02	19	0.15	0.03	**0.002**
MTR	3	26.08	6.88	19	37.38	2.53	**0.005**
QSM [ppm]	2	0.00	0.01	5	0.00	0.00	0.857
R2* [1/s]	2	18.49	1.25	5	18.66	0.72	0.857
qT1 [ms]	2	1086.47	67.92	2	833.92	55.96	0.333

Statistically significant differences are highlighted in bold

Most of the parameters recorded showed significant changes compared to the control group.

In the parameters measured by diffusion-weighted imaging, FA, MD and RD were significantly altered, with FA showing decreased values whilst MD and RD increased values. Mean, axial and radial kurtosis also showed significantly decreased values compared to the control group.

All NODDI-derived parameters (ICVF, ODI, ISO) were significantly different from the control group (all *p* < 0.001 except ODI, which was only slightly less significant with *p* < 0.008). The ICVF and ISO were reduced in the patient group, ODI increased.

MR-spectroscopy data showed significantly lower NAA/Cr values. The values for Cho/Cr did not differ from the controls.

The myelin water fraction was significantly reduced in patients.

Magnetization transfer ratio was significantly reduced in patients compared to controls with *p* < 0.005.

In contrast, QSM, R2* and quantitative T1 values lacked statistical significance. The qT1-values tended to be elevated in patients; however, this difference was not statistically significant (*p* < 0.333).

## Discussion

In the current study, we quantified the volume of T2-hyperintensity in children with the late-infantile form of MLD throughout their disease course, including images from a very advanced disease stage. For the first time, and contraintuitive to what could be expected in T2-weighted images in a leukodystrophy, we demonstrate areas of T2-pseudonormalization in the late stage of the disease, where patients had lost most of their motor and cognitive functions. The additional microstructural MRI parameters obtained by multimodal imaging showed clear and severe microstructural damage, indicating that normalization of the T2-signal in this disease stage does not develop alongside tissue normalization, but instead showing severely pathological tissue microstructure. Therefore, it is suggested to call this phenomenon T2-pseudonormalization.

It can be concluded that for the late-infantile form of MLD T2 changes do not provide a sufficient correlation to the extent and type of tissue damage underlying the clinical symptoms at a very late stage of the disease. To fill this gap in understanding of the disease and monitoring the response to treatment in studies, a combination of more specific imaging parameters might give additional quantitative information about tissue changes, such as advanced diffusion-weighted imaging parameters including diffusional kurtosis or the NODDI model, MR-spectroscopy, myelin water fraction (MWF), magnetization transfer ratio (MTR), effective transverse relaxation rate R2*, T1-mapping and quantitative susceptibility mapping.

Many of the parameters tested here showed significantly different results when compared to the control group. First and foremost, the diffusion imaging parameters including most of the diffusion tensor values, diffusion kurtosis values and NODDI-derived parameters, MWF and MTR should be mentioned in this context, which achieved high effect sizes. In the following, the positive parameters are listed, interpreted and correlated with the literature, if available:

Fractional anisotropy (FA) is decreased in patients, reflecting an increased diffusivity of water molecules than in healthy tissue as an expression of reduced fiber density or myelination. Radial diffusivity (RD), which is considered a correlate of myelin loss [34], is increased in patients. Axonal diffusivity, which was not significantly different between patients and controls, is more likely to be seen as an expression of axonal loss, with both decreased [34] and increased values [35] being associated and therefore deviations should be interpreted with caution. Similar to RD, the mean diffusivity (MD) is increased. The deflection of the changes in FA, MD and RD are identical to the previous DTI studies in MLD [15, 36], in only one study was AD shown to be reduced. The quantitative comparability is limited since van Rappard et al. [15] used different ROIs than in our study.

Diffusion kurtosis as an expression of the deviation from a Gaussian normal distribution is determined by the complexity of the tissue that limits the diffusivity of water molecules. It can, therefore, be regarded as a measure of the degree of the tissue structure [37]. Similar to axial diffusivity, changes in axial kurtosis (AK) are attributed to changes along the main eigenvector, which in white matter corresponds mainly to the orientation of the axons, while radial kurtosis (RK) measures the perpendicular direction and is most likely influenced by compartments such as cell membranes or myelin layers [38, 39]. The decrease of RK and, to a lesser extent, AK in patients agrees with findings that have been reported in mouse models of demyelination in the central nervous system [40, 41].

NODDI-derived parameters allow a further interpretation of the microstructure by assuming a compartmentalization of the tissue [42]. The ICVF is a model for axon and dendrite density [43, 44] and is reduced in patients. The ODI characterizes the angular variation of neurites [45]. The increased value in patients can best be explained as fiber dispersion and thus reduced directionality of axons. The ISO index reflects the proportion of freely diffusing water molecules. The lowered values in patients show a reduced fluid content accordingly.

While the total T2 signal was normal in patients, the T2 distribution arising from myelin water (between 10 and 40 ms for humans in vivo) is highlighted in the myelin water fraction [46]. Lower values for myelin water fraction indicate a reduced myelin content in the measured MLD patients, as would be expected in a demyelinating process. Myelin water fraction has also been shown to be a sensitive but not fully specific parameter for demyelinating processes in other diseases, e.g. multiple sclerosis [47, 48]. It has also been used in another lysosomal storage disease—Niemann Pick—where it also found reduced values in two patients [49], so that MWF might also be a suitable disease parameter for other lysosomal storage diseases. Since MWF was only slightly impaired in post-mortem studies and brains with formalin fixation, MWF is considered relatively insensitive to different water contents [50].

The magnetization transfer ratio is significantly lower in patients. Since myelin in the white matter builds a significant proportion of the macromolecules that are involved in the magnetization transfer effect, this indicates a loss of myelin, as has been repeatedly demonstrated in multiple sclerosis [51, 52].

In MR spectroscopy, only the quotient of N‑acetylaspartate to creatine (NAA/Cr), which is considered a correlate for neuronal integrity [53, 54], was found to be significantly lowered. This result is consistent with previous MR spectroscopy studies in MLD [20, 55, 56]. The choline/creatine peak is a marker of the cellular membrane turnover [57] and did not show changes in our study. In previous studies on MLD patients, Cho/Cr was increased in patients with a good outcome after stem cell transplantation, potentially indicating remyelination [20].

As a comparison to other diseases, the phenomenon of normal appearing T2 signal in the white matter (NAWM) in combination with significant changes in advanced imaging techniques, as used in our study, is described in the context of patients with multiple sclerosis [58–60] and tuberous sclerosis [61]. Changes in diffusion-based parameters, in particular NODDI and myelin water fraction and magnetization transfer ratio, were observed as well. These changes were correlated with disability, cognitive impairment, and the degree of brain atrophy in multiple sclerosis patients and therefore seem to play an important clinical role in the extent of disease symptoms [62]. The reported histopathological samples from such regions show axonal pathology and microglial activation depending on the distance to focal MS lesions [63]. It is unclear to what extent metachromatic leukodystrophy can be deduced from these clinical pictures with different pathomechanisms; however, there is evidence that other disorders of myelination also have some structural pathologies that cannot be reproduced by T2 signal intensity alone.

In connection with the current research question, some parameters remained without evidence of differentiation between patients and healthy controls. While QSM and R2* maps in multiple sclerosis have been shown to be a sensitive disease marker also for different stages of demyelination (acute, advanced, chronic) [62, 63], this cannot be verified in our MLD patients—possibly due to the lack of iron-positive myeloid cells. Also, quantitative T1 values that change with myelination during brain development [64] were not different from healthy controls; however, a limiting factor in the interpretation of the usability of these sequences in the context of metachromatic leukodystrophy is the significantly lower number of healthy controls compared to other sequences studied. Therefore, even these sequences might achieve significant differences compared to a more representative control group.

Taken together, these MR parameters characterize the microstructure in areas with T2-pseudonormalization as a predominantly demyelinating process (indicated by RD and RK, MWF, MTR), but also suggest a loss of axons and fiber structure beyond this (NAA/Cr, AK, FA, ICVF, ODI) as well as very dense tissue without free water molecules (indicated by MD, MK, ISO).

The mechanism of the decrease of the T2 signal can be speculated according to these results but also to histopathological findings reported in MLD patients. Initially, the demyelinating component is predominant due to the accumulation of sulfatides in oligodendrocytes [65], the consequent loss or dysfunction of these lead to myelinolysis. The myelin is replaced by water which causes the T2 signal to increase. As the disease progresses, phagocytes migrate to take on the lost cells and lipid deposits; however, since the phagocytes are also deficient in arylsulfatase A, they accumulate non-degradable components themselves and eventually die [1]. A vicious circle develops with ever-increasing lipid-containing bodies and cell decay products as well as gliosis [66]. These structures, densely packed in the late stage of the disease, displace water to such an extent that a drop in the T2 signal occurs. Substantial parts of the structures may consist of sulfatides, since these lipids are highly polar which allows them to assemble as micelles, with the sulfate moieties grouped as polyanionic surfaces on aggregates of the micelles that are responsible for the strong metachromasia in the disintegrated, condensed white matter on histochemistry [67]. Correspondingly, the consistency of the white matter was described in autopsies as “firmer than usual” [68] and “hard in consistency” [69].

However, it should be noted that the mechanisms of T2 normalization cannot be conclusively clarified in the absence of direct histopathological examination of tissues of the examined patients.

Limitations of the current study are the small number of cases, the different sizes of the control groups and especially the small group size in QSM, R2* and qT1map, the asymmetric age of the control groups compared to patients and the lack of follow-up for the more advanced sequence parameters over the course of the disease. Furthermore, data collection was carried out in favor of the feasible scanning time in only 1 ROI. To increase the spatial extent of measurements, future studies might refrain from collecting the parameters that we found to be non-significant.

However, it can be stated based on the available data that imaging extended by these parameters is useful in MLD patients in order to better quantify the extent of affected tissue, to better understand pathomechanisms and the resulting symptoms in the course of the disease, and to provide more reliable markers for therapy studies than T2 values, which, as described here, can also deceptively indicate normal values despite severe alterations, which could also be misinterpreted as a positive response to treatment. To a certain extent, this can probably also be transferred to the other forms of MLD (juvenile, adult) or other leukodystrophies with available therapeutic approaches, such as Krabbeʼs disease.

## Conclusion

We found a pseudonormalization of the disease-characteristic T2-signal changes in the late stage of late-infantile MLD. In the context of treatment trials in which MRI serves as an objective parameter for the course of the disease, this phenomenon could erroneously be interpreted as a therapeutic response. In addition, the T2 signal does not provide sufficient information about microstructural damage corresponding to a pathomechanism in MLD that goes beyond demyelination processes. We therefore recommend additional sequences in measurement protocols that can detect microstructural damage despite pseudonormalization. In particular, the diffusion weighted parameters MD, RD, the NODDI-derived parameters ICVF, ODI and ISO, the diffusion kurtosis parameters AK, RK and MK, MWF and MTR as well as MR-spectroscopy for NAA/Cr have been shown to be highly significant markers.

## Abbreviations

AD: Axial diffusion
AK: Axial kurtosis
Cho/Cr: Quotient of choline/creatine peak
CS: Centrum semiovale
DTI: Diffusion tensor imaging
DWI: Diffusion weighted imaging
FA: Fractional anisotropy
ICVF: Intracellular volume fraction
ISO: Isotropic volume fraction
MD: Mean diffusion
MK: Mean kurtosis
MLD: Metachromatic leukodystrophy
MPRAGE: Magnetization-prepared rapid acquisition gradient echo
MP2RAGE: Magnetization-prepared 2 rapid acquisition gradient echos
MS: Multiple sclerosis
MTR: Magnetization transfer ratio
MWF: Myelin water fraction
NAA/Cr: Quotient of N‑acetylaspartate to creatine peak
NODDI: Neurite orientation dispersion and density imaging
ODI: Orientation dispersion index
R2*: Effective transverse relaxation rate
RD: Radial diffusion
RK: Radial kurtosis
QSM: uantitive susceptibility maps
WM: White matter
